# MicroRNA-206: Effective Inhibition of Gastric Cancer Progression through the c-Met Pathway

**DOI:** 10.1371/journal.pone.0128751

**Published:** 2015-07-17

**Authors:** Zhiqiang Zheng, Dongsheng Yan, Xiaoyan Chen, He Huang, Ke Chen, Guangjing Li, Linglin Zhou, Dandan Zheng, LiLi Tu, Xiang Da Dong

**Affiliations:** 1 Wenzhou Medical University, Wenzhou, Zhejiang, China; 2 The Second Affiliated Hospital, Wenzhou Medical University, Wenzhou, Zhejiang, China; 3 School of Ophthalmology and Optometry, Eye Hospital, Wenzhou Medical University, Wenzhou, Zhejiang, China; 4 State Key Laboratory Cultivation Base and Key Laboratory of Vision Science, Ministry of Health of P. R. China, Zhejiang Provincial Key Laboratory of Ophthalmology and Optometry, Wenzhou, Zhejiang, China; 5 Department of Surgery, Stamford Hospital—Affiliate of Columbia University, Stamford, Connecticut, United States of America; Sapporo Medical University, JAPAN

## Abstract

MicroRNAs are endogenous short chain nucleotide RNAs that regulate gene function by direct binding of target mRNAs. In this study, we investigated the effects of microRNA-206 (miR-206) on the development of gastric cancer. miR-206 was first confirmed to be downregulated in gastric cancer specimens. Conversely, upregulation of c-Met was confirmed in tissue samples of human gastric cancer, with its level inversely correlated with miR-206 expression. Introduction of miR-206 inhibited cellular proliferation by inducing G1 cell cycle arrest, as well as migration and invasion. Moreover, important proliferation and/or migration related molecules such as c-Met, CDK4, p-Rb, p-Akt and p-ERK were confirmed to be downregulated by Western blot analysis. Targeting of c-Met also directly affected AGS cell proliferation, migration and invasion. *In vivo*, miR-206 expressing tumor cells also displayed growth delay in comparison to unaffected tumor cells. Our results demonstrated that miR-206 suppressed c-Met expression in gastric cancer and could function as a potent tumor suppressor in c-Met overexpressing tumors. Inhibition of miR-206 function could contribute to aberrant cell proliferation and migration, leading to gastric cancer development.

## Introduction

Gastric cancer is the fourth most common cancer and the second leading cause of cancer related death in the world [1]. Its morbidity and mortality is particularly pronounced in Asian countries due to a variety of influences [1]. Since its presentation is often associated with advanced disease, there is an urgent need for advances in its detection and ultimately its management. At present, the understanding of microRNAs(miRNAs) on influencing gastric cancer formation is occurring at a rapid pace.

Since the first description of miRNA in the nematode *C*. *elegans* back in 1993, the impact of these small non-coding RNAs has transcended multiple branches of molecular biology [2]. MiRNAs are highly tissue specific biomarkers with the potential to alter and transform resident tissue. Because overexpression and under-expression have both been associated with tumorigenesis [3], their roles as oncogenes and tumor suppressor genes are both well-established [4, 5]. Over the last several years, their impact on development and detection of solid organ tumors including gastric cancer is slowly being elucidated. There are already several miRNAs identified in the gastric cancer anti-apoptotic mechanism such as miR-21 and miR-148a [6, 7]. Other pathways influenced by miRNAs include cell cycle progression comprising of miR-222/221, miR-106b/93/25 and miR-24 [6, 7].

One of the other promising new miRNAs for solid organ tumors includes miR-206 [8]. This particular miRNA belongs to a group of “myomiRs” that is involved in skeletal muscle development [9]. Having been associated with numerous other diseases including heart disease, chronic obstructive pulmonary disease and Alzheimer’s, its role in oncogenesis received scrutiny more recently including rhabdomyosarcoma, lung cancer, colorectal cancer, schwannoma, and gastric cancer [8, 9]. Although elevated in a few types of cancer including ovarian and Waldenstrom macroglobulinemia, miR-206 is mostly suppressed in solid organ tumors [9]. miR-206 has previously been shown to inhibit gastric cancer proliferation in part by suppressing cyclin D2 [10]. In this investigation, we concentrated on the role of miR-206 in gastric cancer oncogenesis through the c-Met pathway, which has traditionally been an influential signaling pathway for oncogenesis in a variety of tumors [11]. c-Met has been predicted and shown to be the target gene of multiple miRNAs including miR-206 [9, 12].

## Results

### Suppression of miR-206 led to increased c-Met expression in gastric cancer

**Fig 1:**
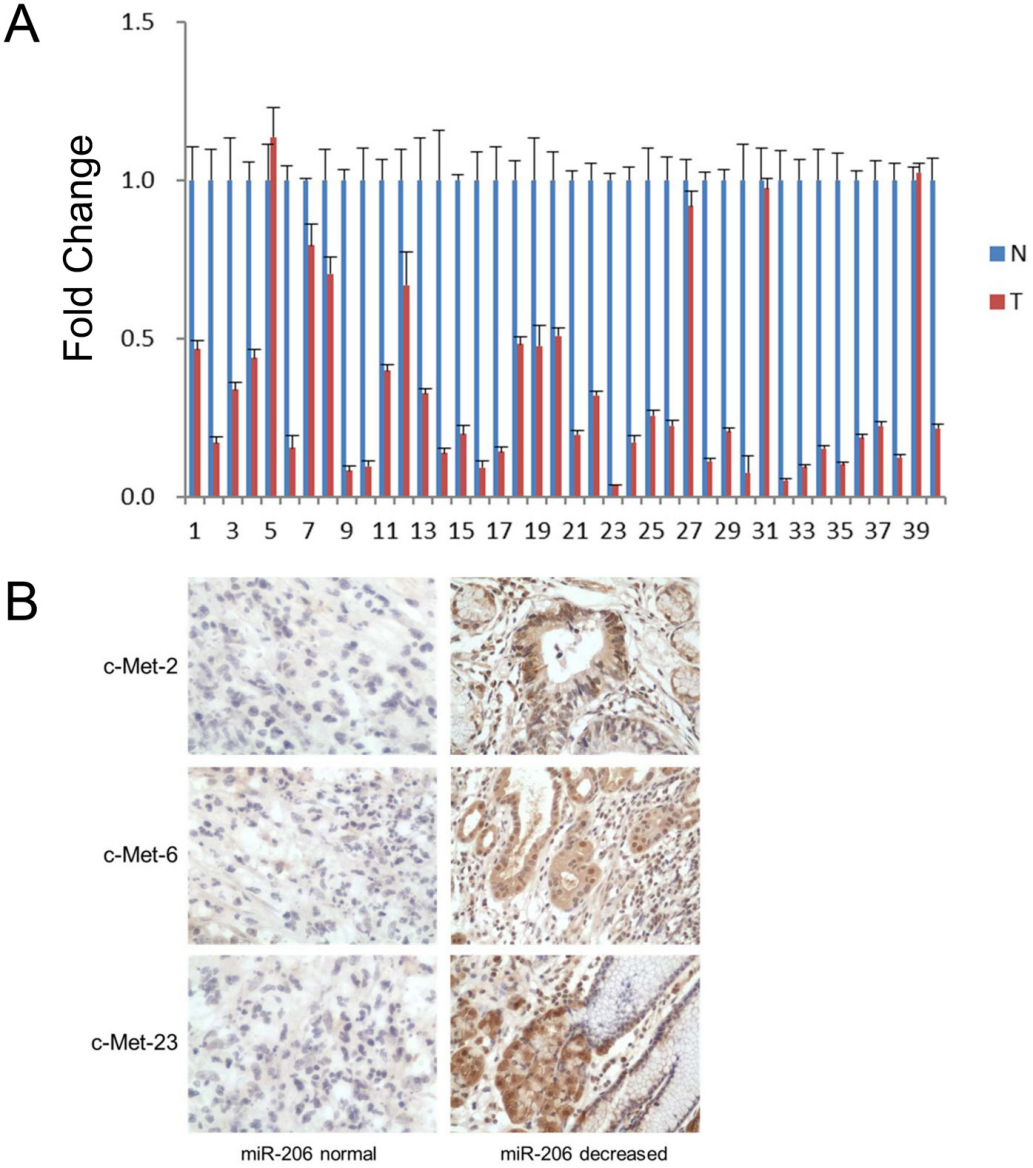
miR-206 expression is associated with weak c-Met expression in gastric tumors. (A) Real-time RT-PCR analysis showing the expression of miR-206 in normal tissues (set at 1) and the relative amount of miR-206 in the tumors, as fold reduction. N: normal tissues; T: tumors. U6 snRNA was used as an internal control. (B) miR-206 expression in cells was inversely correlated with c-Met. The representative immunohistochemical staining of three gastric tumor specimens and their respective adjacent normal tissues was presented. Sample numbers 2, 6, and 23 are identical with those in Real-time RT-PCR. Tumor cells with > 50% positive staining were considered to have strong c-Met expression.

Real-time RT-PCR analysis was performed to detect the expression of miR-206 in 40 gastric cancer specimens and normal tissues. miR-206 levels in most tissue samples of gastric tumor (34/40) were found to be significantly lower than normal tissues (Fig 1A). miR-206 expression was inversely related to the level of c-Met observed in tumor samples (Fig 1B). Most tumor samples, with decreased miR-206 expression, showed high percentage (>50%) of c-Met staining. Conversely, tumors with normal expression of miR-206 showed very weak or negative c-Met expression.

### miR-206 induced G1 arrest and inhibited cell proliferation, migration and invasion of AGS gastric cancer cells

**Fig 2:**
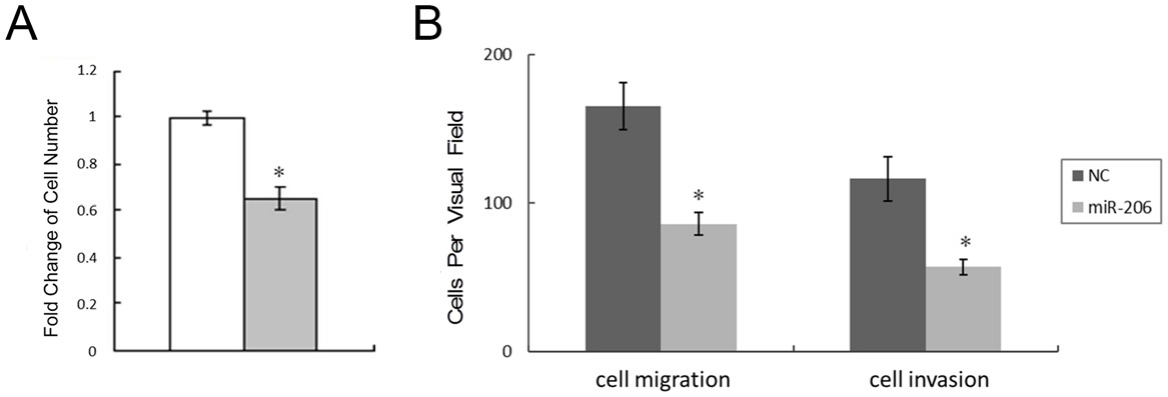
Ectopic miR-206 induces G1 arrest and inhibits cell proliferation, migration and invasion. (A) MTS cell proliferation assay was carried out on day 3 as indicated. (B) AGS cells were assessed with Transwell and Matrigel assays. The number of cells that had migrated through the culture insert pores (left) or had invaded through the Matrigel insert pores (right) was quantified by counting five independent visual fields. *: Differences in cell migration or invasion between miR-206 and negative control transfected cells were significant, *P* < 0.01.

As miR-206 expression was decreased in gastric cancer specimens, we sought to determine whether the introduction of miR-206 had any biological effect on AGS cells. AGS cells transfected with the miR-206 molecule showed inhibition of cell growth as compared to negative control based on the MTS assay (Fig 2A). FACS analysis of the cells showed G1 cell cycle arrest (S1 Fig). The number of colonies was also reduced with transfection of miR-206 (S2 Fig).

miR-206 can inhibit migration and invasion of AGS cells (Fig 2B and S3 Fig). A dramatic reduction of migration towards the lower chambers was observed in miR-206 transfected AGS cells (86 ± 15 vs. 165 ± 16 in AGS cells, *P* < 0.01, n = 3). In addition, cells transfected with miR-206 showed that HGF-induced invasiveness was also significantly hampered following miR-206 transfection (57 ± 12 vs. 116 ± 14 in AGS cells, *P* < 0.01, n = 3).

### miR-206 downregulated c-Met expression and other cell cycle-related proteins

**Fig 3:**
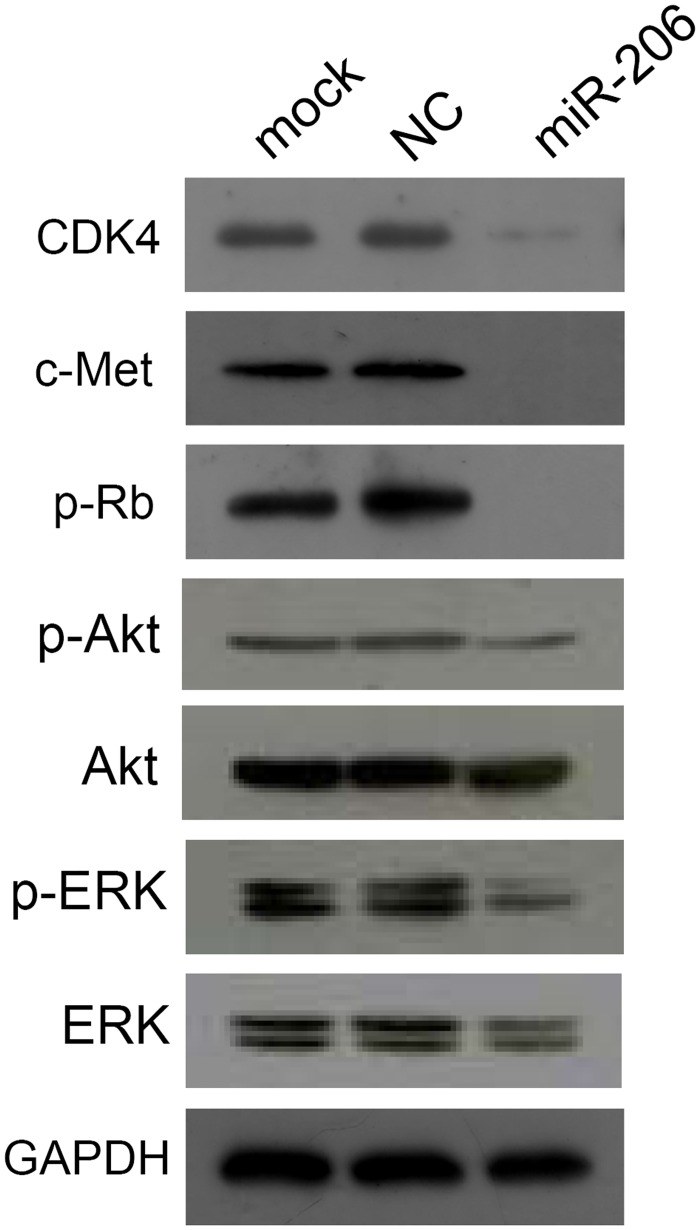
miR-206 downregulates the expression of c-Met, cycle-related proteins CDK4, and phosphorylated-Rb (p-Rb) in AGS cells. miR-206 also downregulates expression of p-Akt and p-ERK1/2, but not total Akt or ERK1/2.

We have previously identified c-Met as a direct target of miR-206 [9]. Western blot analysis confirmed that c-Met expression was reduced by miR-206 transfection in AGS cells (Fig 3). Concurrently, ectopic miR-206 also down-regulated the expression of CDK4, p-Rb, p-Akt and p-ERK.

### Downregulation of c-Met inhibited gastric cancer cell proliferation, migration and invasion

**Fig 4:**
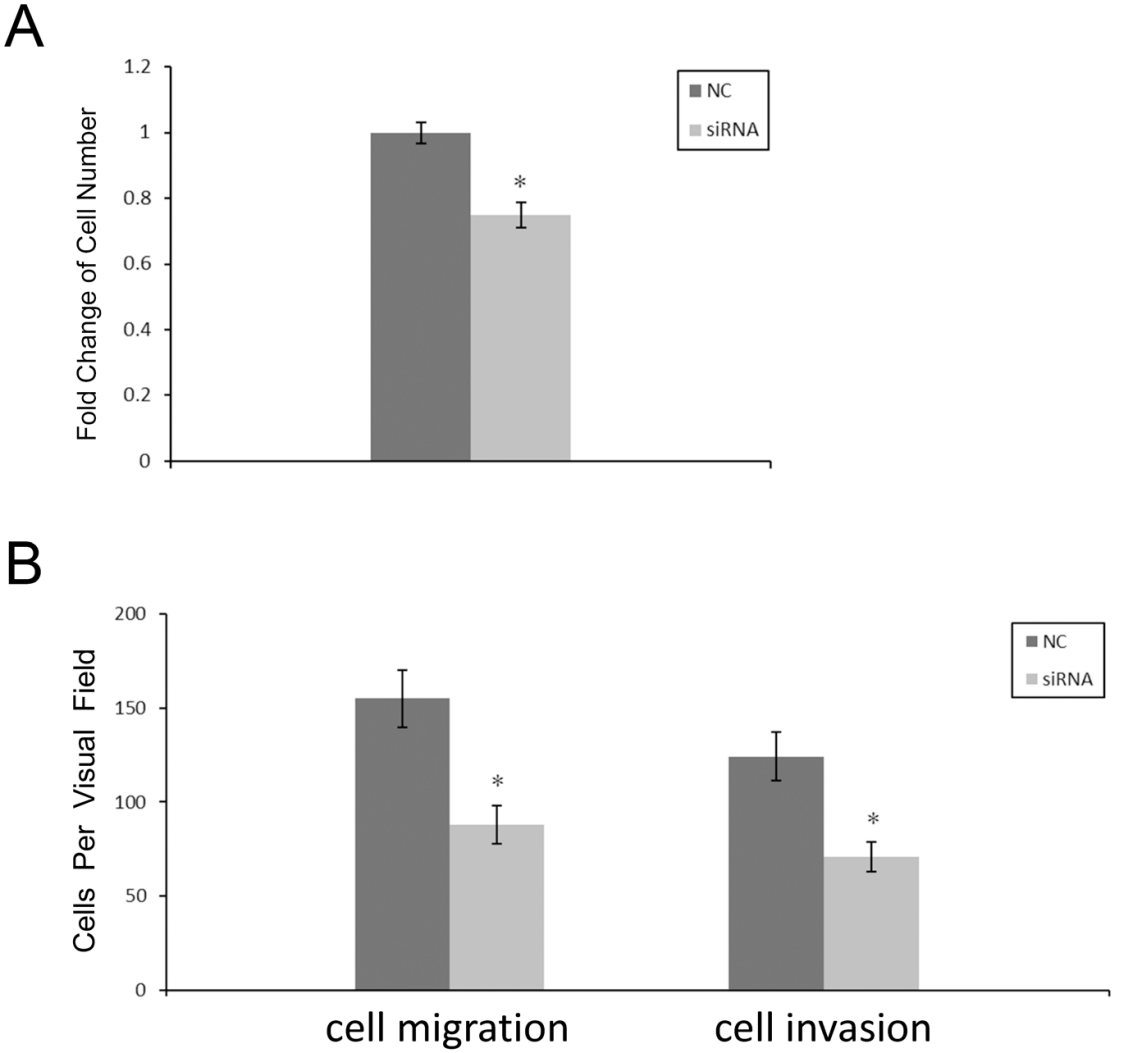
Downregulation of c-Met inhibits AGS cell proliferation, migration and invasion. (A) MTS cell proliferation assay was carried out on day 3 after transfection. (B) The impact of c-Met siRNA on AGS cells was quantified using culture or Matrigel inserts.

Next, c-Met specific siRNA was first used to decrease the expression of c-Met in AGS cells (S4 Fig). MTS assays were performed to detect the proliferation of cells. AGS cells transfected with c-Met siRNA showed reduced cell growth as compared to negative control cells (Fig 4A; 25.10 ± 3.81% decrease). Both HGF-induced migration and invasion were decreased when comparing c-Met siRNA transfected cells to negative control transfected cells. As indicated in Fig 4B and S5 Fig, decrease in migration (88 ± 10 vs. 155 ± 15, P < 0.01, n = 3) and invasion (72 ± 7 vs. 126 ± 12, P < 0.01, n = 3) were both statistically significant.

### Introduction of miR-206 suppressed tumor growth *in vivo*

**Fig 5:**
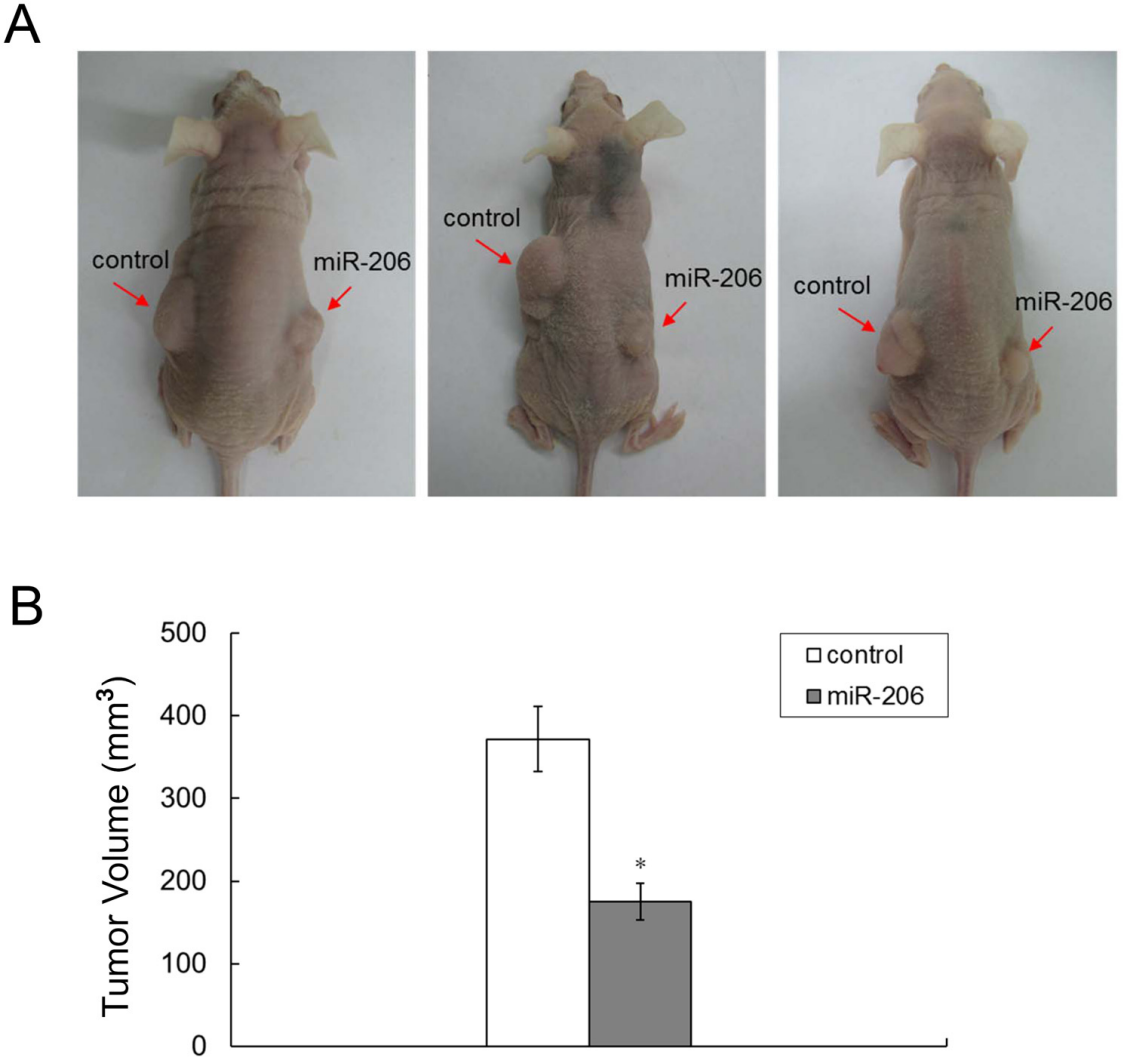
Introduction of miR-206 in AGS cells suppresses tumor growth in nude mice. (A) Representative photographs of nude mice 8 weeks after inoculation. (B) Average volume of tumors was statistically significant. *: n = 5 each, *P* < 0.01.

We next investigated if overexpression of miR-206 could repress tumor growth *in vivo*. After 8 weeks, the averaged tumor volumes were significantly lower from cells infected with lentivirus expressing miR-206, as compared with control (Fig 5).

## Discussion

Gastric cancer has remained a significant health care burden worldwide despite years of research [1]. According to the WHO, gastric cancer remains one of the top cancer etiologies with a high rate of mortality [1]. As with other solid organ tumors, it is increasingly clear that better understanding of tumor oncogenesis is necessary to improve the prognosis of these patients. Being prevalent in Asian countries, data are emerging on the role of miRNAs on the development or suppression of gastric cancers [6].

miRNAs have dual functions in terms of gastric cancer development [6, 13]. Some miRNAs are tumor suppressors and others are oncomiRs [6]. Ueda et al. found 22 miRNAs upregulated and 13 downregulated in the plasma of patients with gastric cancer [13]. Targets that are under investigation in the case of gastric cancer include regulators of p16, via miR-24, and p21 through miR-222/221 and miR-106b/93/25 [6]. Others, such as miR-21, can be upregulated in up to 92% of gastric cancer tissue samples [14]. Candidates identified in gastric cancer serving as tumor suppressors include miR-181b, miR-101 and miR-486 [6]. For instance, miR-101 is thought to target EZH2, Cox-2, Mcl-1 and Fos [15]. miR-486 is thought to affect OLFM4, possibly affecting apoptosis downstream [16].

Having established that the majority of miRNAs serve as tumor suppressors, we investigated the c-Met pathway in gastric cancer. In studying the c-Met pathway for rhadbomyosarcoma, we found the importance of this pathway in sarcoma [9]. c-Met is known to be dysregulated in gastric cancer [11, 17–19]. The MET proto-oncogene encodes a protein known as hepatocyte growth factor (HGF) receptor which possesses tyrosine kinase activity [18]. We normally find it expressed only in stem cells but have also seen its dysregulation in oncogenesis [18]. In this study, we were able to confirm that c-Met is significantly involved in gastric cancer and its role as a miR-206 target is pivotal in oncogenesis.

Cell cycle progression is dysregulated in gastric cancer. Based on previous reports, cyclin D2 is elevated in gastric cancer when miR-206 is affected [10]. miR-206 has been shown to be a potent prognostic marker [10]. Its downregulation is associated with shorter overall survival. Restoration of miR-206 leads to G0/G1 cell cycle arrest, confirming its role as a tumor suppressor. Our work also showed cell cycle dysregulation with CDK4 and phosphorylated-Rb being affected. CDK4 is a member of the cycline dependent kinase family with ser/thr protein kinase activity. It leads to G1 phase progression. In addition, the kinase leads to the phosphorylation of Rb, which is a tumor suppressor involved in cell cycle progression.

Although similar findings have been confirmed in gastric, ovarian and breast cancers, the role of miR-206 seems to have an opposite effect in colon cancer [6, 20]. An inverse correlation was noted between miR-206 and KLF4 in a panel of human colon cancers [20]. KLF4, which has oncogenic properties in other cancers such as breast, skin and lung, functions as a tumor suppressor in colon cancer [20]. Its promoter is hyper-methylated with elevated levels of miR-206 leading to downregulation of KLF4 [20]. These findings indicate that miR-206 may not function similarly in all epithelial cells, which negates the one size fits all approach in chemotherapeutics.

In the present study, we identified a mechanism for regulation of c-Met gene expression through miR-206 in gastric cancer. c-Met overexpression following miR-206 downregulation seems to be the common etiology for the pathogenesis of gastric cancer in the majority of samples examined in this study. In summary, we demonstrated that miR-206 negatively modulates the c-Met signaling pathway involved in cell proliferation and migration. Our studies will hopefully have important clinical consequences in the treatment of gastric cancer. miR-206 is a candidate for both immunohistochemical detection of small tumors and possible target for biological therapeutics.

## Materials and Methods

### Cell culture

The human gastric cancer cell line, AGS, purchased from ATCC (Manassas, VA), was grown in Ham's F-12 Medium (Invitrogen, Carlsbad, CA) supplemented with 10% fetal bovine serum (FBS; Hyclone, Logan, UT).

### Ethics statement

This study was carried out in strict accordance with the recommendations and approval of the Wenzhou Medical University Animal Care and Use Committee (Permit Number: WZMCOPT-043011). Forty gastric tumor specimens and normal donor gastric tissues were obtained from the second affiliated hospital of Wenzhou Medical University (Wenzhou, China). Sample collection was approved by the Wenzhou Medical University Ethics Committee on research involving human subjects, and written informed consent was obtained from each case. All experiments were performed in compliance with the Helsinki Declaration and national laws.

### Quantitative RT-PCR

Total RNA was extracted from human gastric tumor samples and normal controls with Trizol reagent (Invitrogen). 10 ng of total RNA were used for cDNA synthesis by the Taqman MicroRNA Reverse Transcription Kit (Applied Biosystems, Foster City, CA), and miR-206 expression level was quantified by the Taqman MicroRNA Assay (Applied Biosystems). Real-time RT-PCR was performed using the Applied Biosystems ViiA 7 Real-Time PCR System (Applied Biosystems).

### Immunohistochemical staining of c-Met

Sections of formalin-fixed paraffin-embedded human gastric tumor specimens (5 μm) were made and then de-paraffinized with xylene and ethanol. Slides were treated with 0.3% hydrogen peroxide and then incubated overnight at 4°C with c-Met antibody at 1:300 dilution (CST, Beverly, MA). Immunohistochemical staining was performed using the EnVision HRP/DAB detection system (Dako, Glostrup, Denmark).

### Cell proliferation assay

AGS cells were plated at 2000 cells per well in 96-well plates (Costar, High Wycombe, UK) for each transfection. Transfections were performed with reagent (Lipofectamine RNAiMAX; Invitrogen), in triplicates. For each well, 50 nM miR-206 mimics molecule (Ambion, Austin, TX), or a negative control (Ambion) transfection was employed. After 72 hours of culture, cell proliferation was assessed by MTS assay (CellTiter 96 AQueous; Promega, Madison, WI).

### Transwell migration assays

24 hours following transfection, AGS cells were harvested by trypsinization and washed once with D-Hanks solution (Invitrogen). To measure cell migration or invasion, 8-μm pore size culture or Matrigel inserts (Transwell; Costar) were placed into the wells of 24-well culture plates. In the lower chamber, 400 μL F-12 containing 10% FBS and 20 ng/ml of HGF was added. Then, 5x10^4^ cells were added to the upper chamber. After 20 hours of incubation, the cells that had migrated through the pores were stained with crystal violet and observed under the microscope (Zeiss, Oberkochen, Germany) using a 20X objective.

### Western blot analysis

AGS cells (1x10^5^) were seeded in 6-well plates and grown in F-12 for 24 hours prior to transfection. 72 hours after transfection, the cells were washed with cold PBS and subjected to a lysis buffer (35 mM Tris-Cl [pH 6.8], 20 g/L sodium dodecyl sulfate [SDS], 100 mM dithiothreitol). Protein lysates (20 μg each) were separated using 8% SDS-polyacrylamide gel electrophoresis, then electro-transferred onto nitrocellulose filter membranes. The membranes were blocked with a buffer containing 5% nonfat milk in PBS with 0.05% Tween-20 for 2 hours and incubated overnight with antibody at 4°C. After a second wash with PBS containing 0.05% Tween-20, the membranes were incubated with peroxidase-conjugated secondary antibodies (Millipore, Darmstadt, Germany) and developed with an enhanced chemiluminescence detection kit (Pierce, Rockford, IL). GAPDH was used as a loading control. Antibodies for CDK4, p-Rb, ERK, Akt, p-ERK, p-Akt, c-Met and GAPDH were from Cell Signaling Technology (Beverly, MA).

### SiRNA assays

c-Met-specific siRNA (Ambion) and negative control siRNA (Ambion) were used to downregulate c-Met expression in AGS cells. 50 nM of c-Met-specific siRNA or negative control siRNA was transfected into AGS cells with Lipofectamine RNAiMAX. MTS assay was carried out 72 hours after transfection, whereas Transwell and Matrigel assays were performed 24 hours after transfection, as described above.

### *In vivo* tumor growth assay

The pre-microRNA expression constructs lenti-miR-206 and pCDH-CMV-MCS-EF1-copGFP control vector were purchased from System Biosciences (Mountain View, CA). AGS cells were infected with lentivirus expressing miR-206 or negative control. Female nude mice, 6 weeks of age, were inoculated with AGS cells (8x10^6^) expressing miR-206 or negative controls in their flanks, and then sacrificed after 8 weeks. Tumor size was measured and volume was calculated using the formula: (*L* x *W* ^2^) x 0.5, (*L*, length; *W*, width), according to the method previously reported [21]. All studies and procedures were approved by the Wenzhou Medical University Animal Care and Use Committee.

### Statistical analysis

All data were shown as the mean ± SEM. Results shown are expressed as the mean value ± SEM of the results obtained from triplicates in one experiment. Results represent those obtained in three separate experiments. Differences between experimental groups and control groups were analyzed using the Student’s *t*-test. Statistical significance was accepted at *P* < 0.05.

## Supporting Information

S1 Fig FACS analysis of AGS cells transfected with miR-206. AGS cells were collected 48 hours after transfection with miR-206 or NC, stained with propidium iodide, and analyzed by flow cytometry. Ten thousand cells were evaluated in each sample. The most representative results in three independent experiments are depicted.
(TIF)

S2 Fig Colony formation assay. AGS cells transfected with miR-206 or NC were seeded at low density. After 7 days, colony formation was determined by staining with crystal violet. Typical results in three independent experiments are shown.
(TIF)

S3 Fig The effects of miR-206 on AGS cells were assessed with Transwell and Matrigel assays. The number of cells that had migrated through the culture insert pores (up) or had invaded through the Matrigel insert pores (down) was photographed using a 20X microscope objective.
(TIF)

S4 Fig Downregulation of c-Met by siRNA. Western blot analysis was performed to confirm suppression of c-Met expression after lipofectamine transfection of AGS cells with either c-Met siRNA or a negative control (NC).
(TIF)

S5 Fig The effects of c-Met siRNA on AGS cells were assessed with Transwell and Matrigel assays. The number of cells that had migrated through the culture insert pores (up) or had invaded through the Matrigel insert pores (down) was photographed using a 20X microscope objective.
(TIF)
