# Supplementary figures and images for: MicroRNA-206: Effective Inhibition of Gastric Cancer Progression through the c-Met Pathway
Source: PLoS One. 2015 Jul 17;10(7):e0128751. doi: 10.1371/journal.pone.0128751 (PMC4505964; doi:10.1371/journal.pone.0128751)

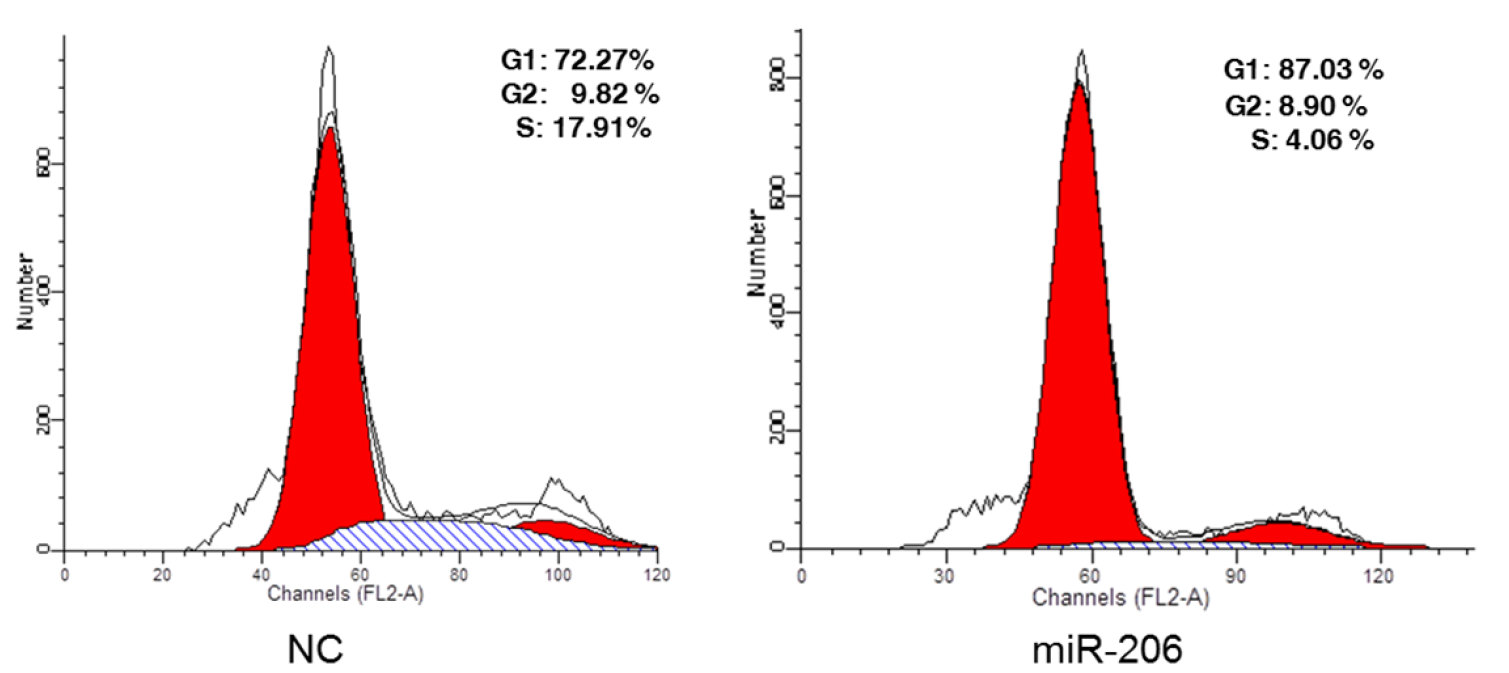

Supplement: S1 Fig — AGS cells were collected 48 hours after transfection with miR-206 or NC, stained with propidium iodide, and analyzed by flow cytometry. Ten thousand cells were evaluated in each sample. The most representative results in three independent experiments are depicted. (TIF) [file pone.0128751.s001.tif]

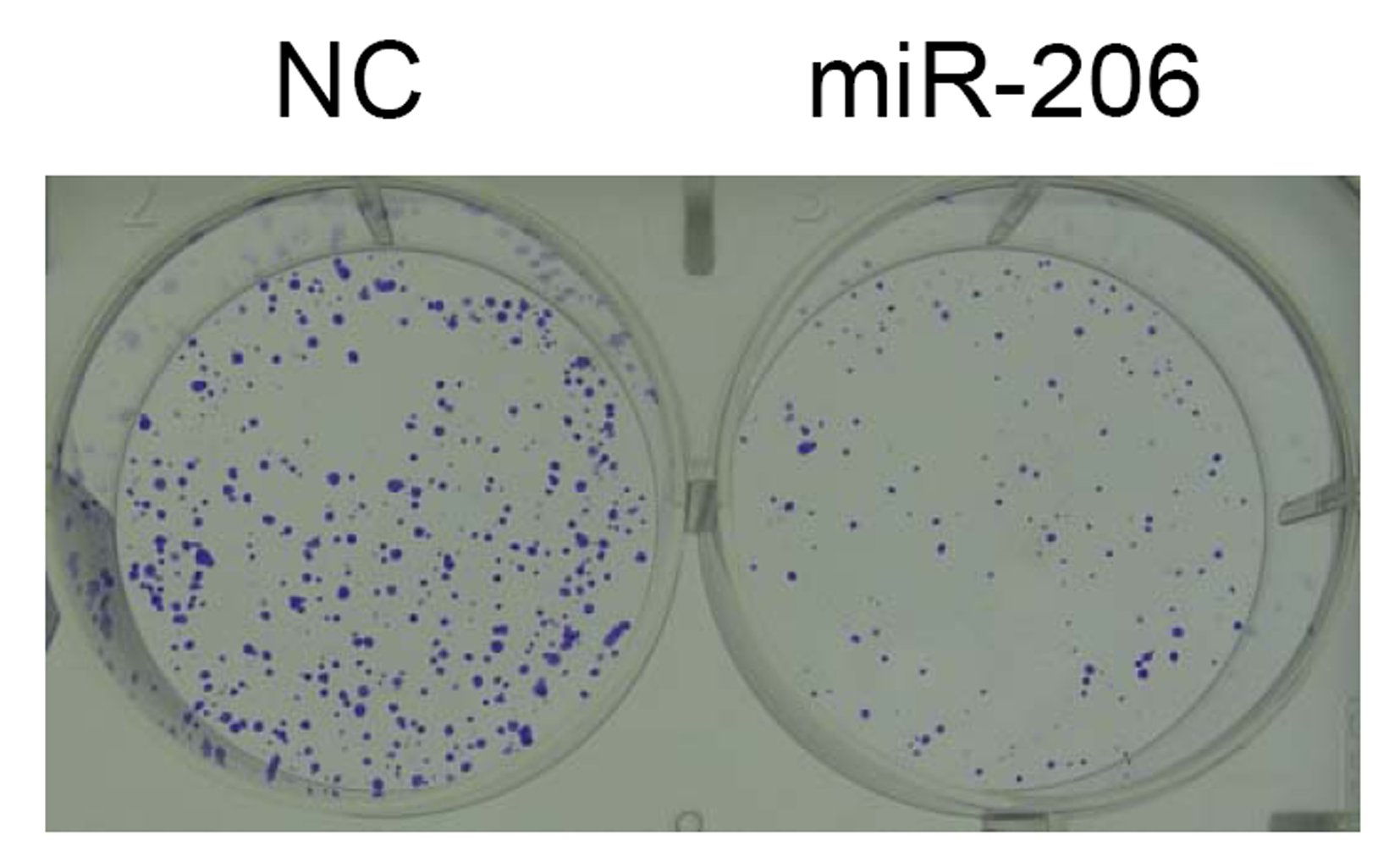

Supplement: S2 Fig — AGS cells transfected with miR-206 or NC were seeded at low density. After 7 days, colony formation was determined by staining with crystal violet. Typical results in three independent experiments are shown. (TIF) [file pone.0128751.s002.tif]

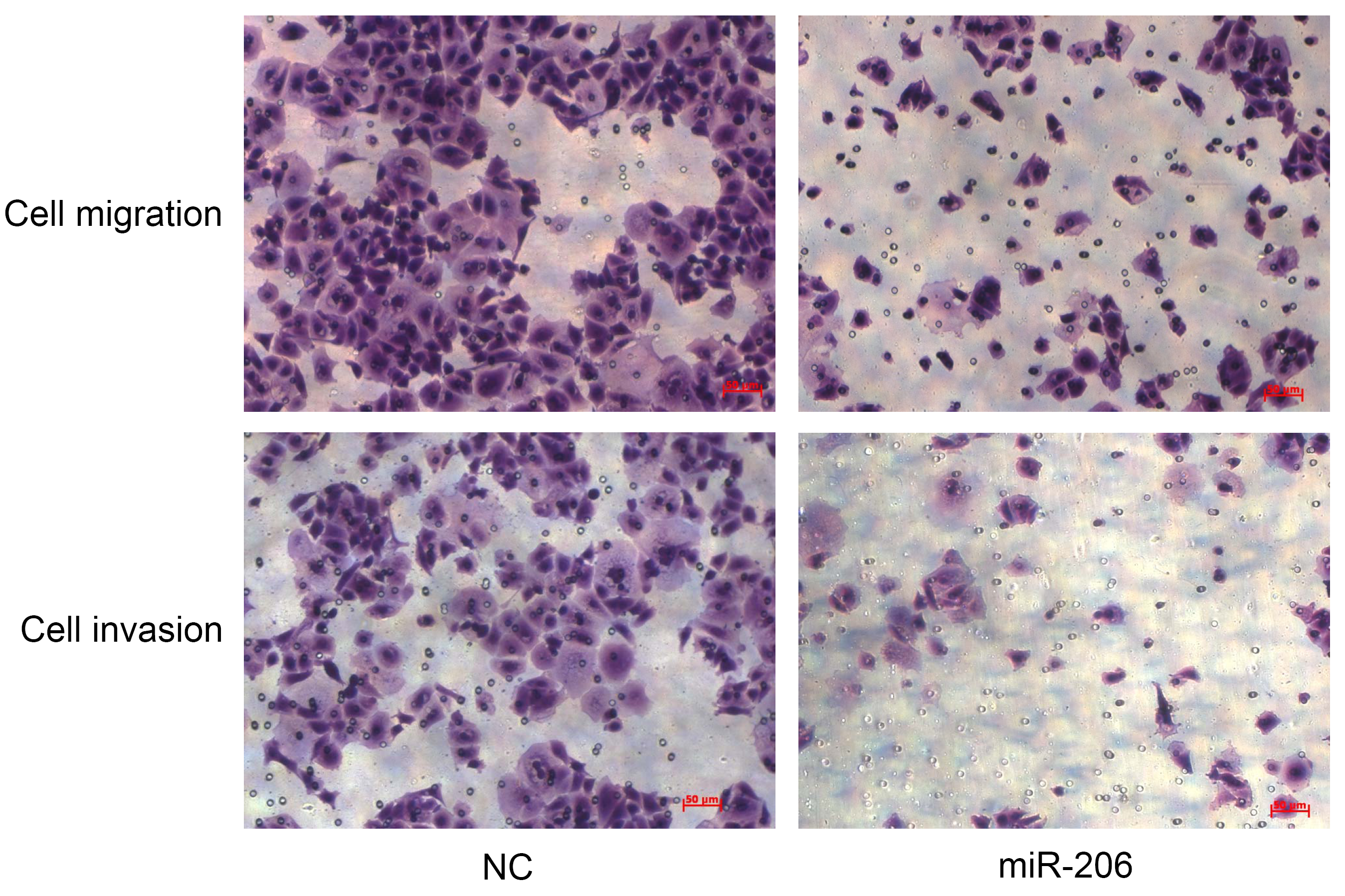

Supplement: S3 Fig — The number of cells that had migrated through the culture insert pores (up) or had invaded through the Matrigel insert pores (down) was photographed using a 20X microscope objective. (TIF) [file pone.0128751.s003.tif]

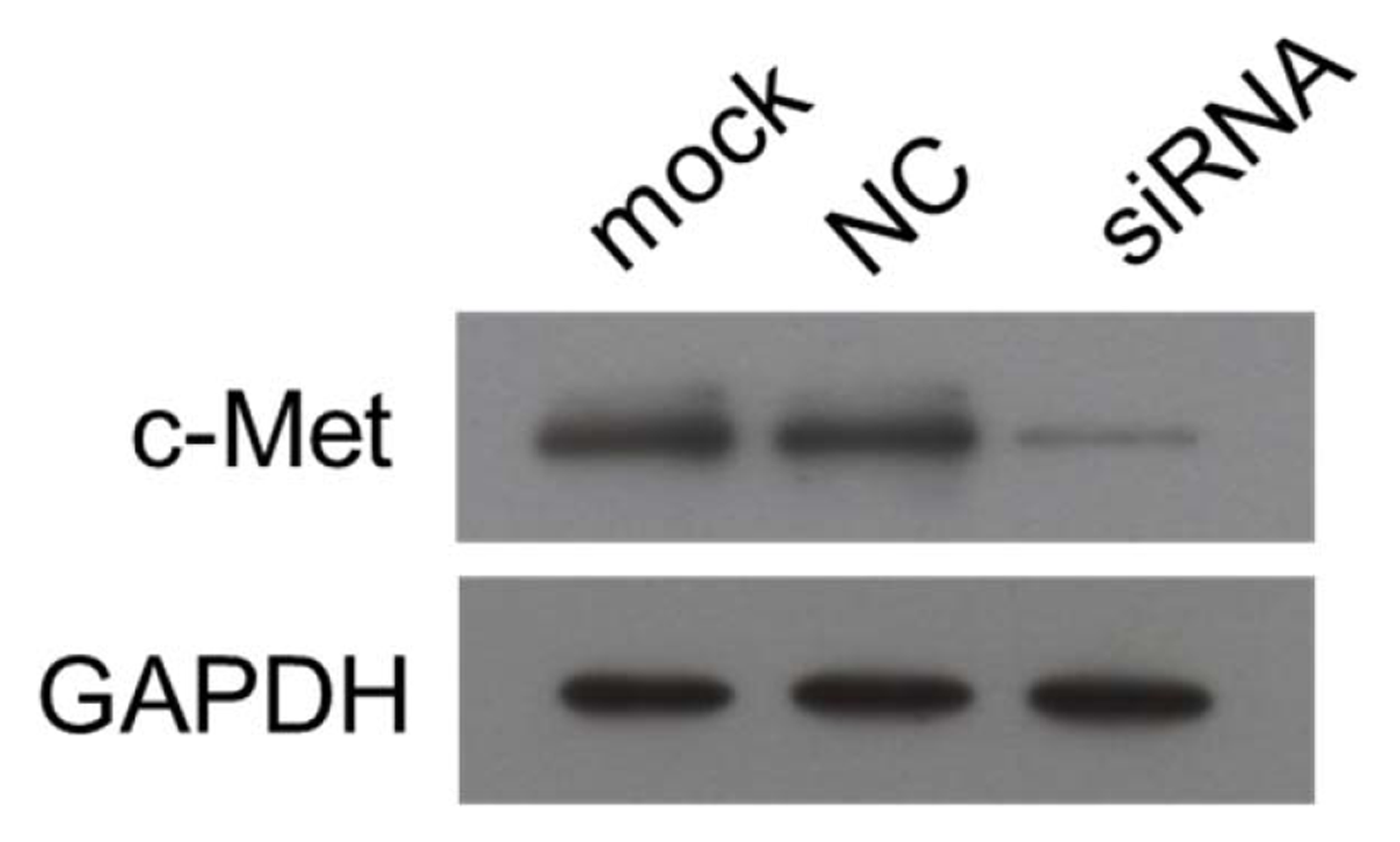

Supplement: S4 Fig — Western blot analysis was performed to confirm suppression of c-Met expression after lipofectamine transfection of AGS cells with either c-Met siRNA or a negative control (NC). (TIF) [file pone.0128751.s004.tif]

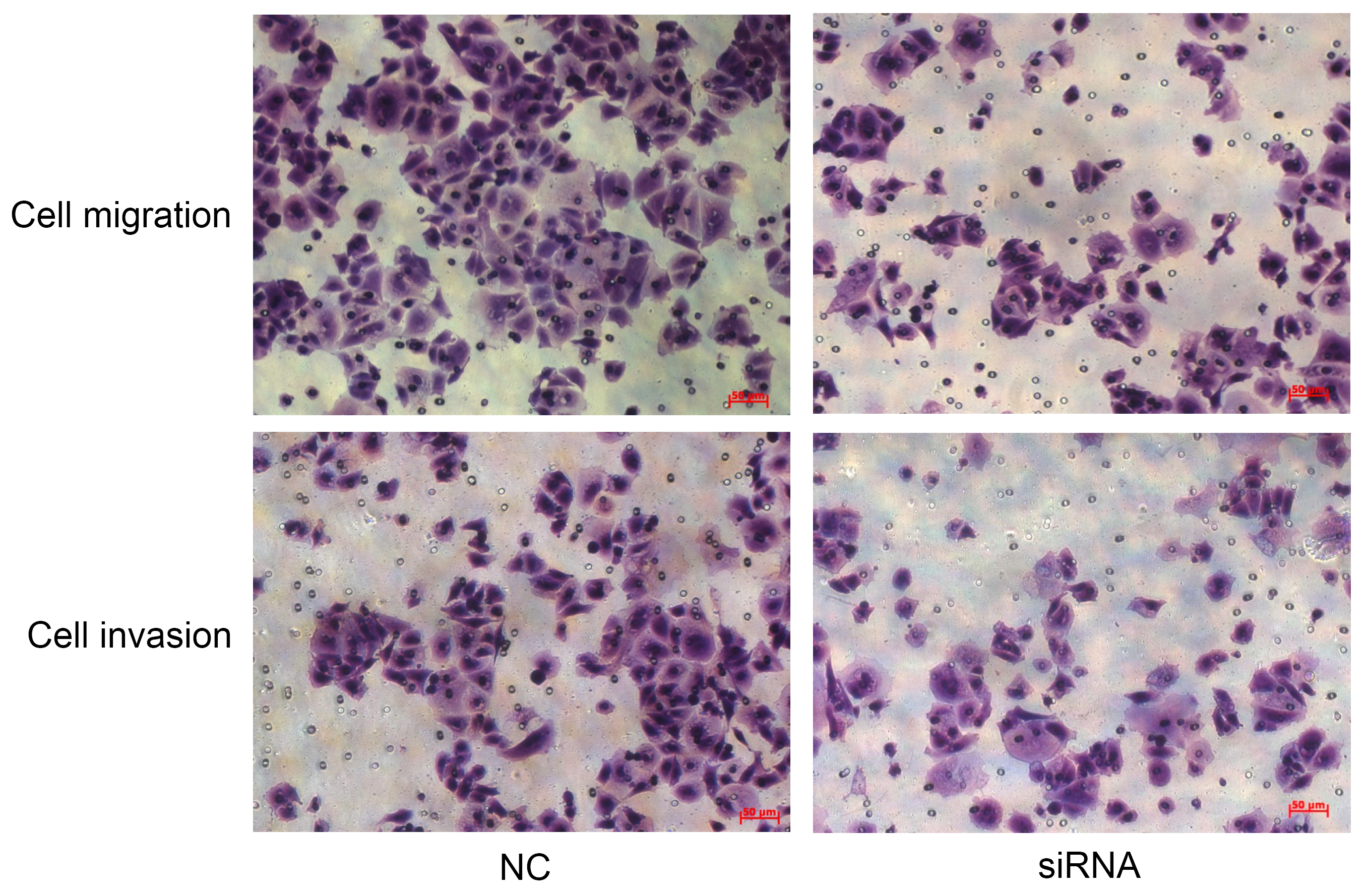

Supplement: S5 Fig — The number of cells that had migrated through the culture insert pores (up) or had invaded through the Matrigel insert pores (down) was photographed using a 20X microscope objective. (TIF) [file pone.0128751.s005.tif]
